# Supplementary figures and images for: Identification and Characterization of ERK2 Dimerization Inhibitors by Integrated In Silico and In Vitro Screening
Source: Int J Mol Sci. 2025 Nov 27;26(23):11481. doi: 10.3390/ijms262311481 (PMC12692606; doi:10.3390/ijms262311481)

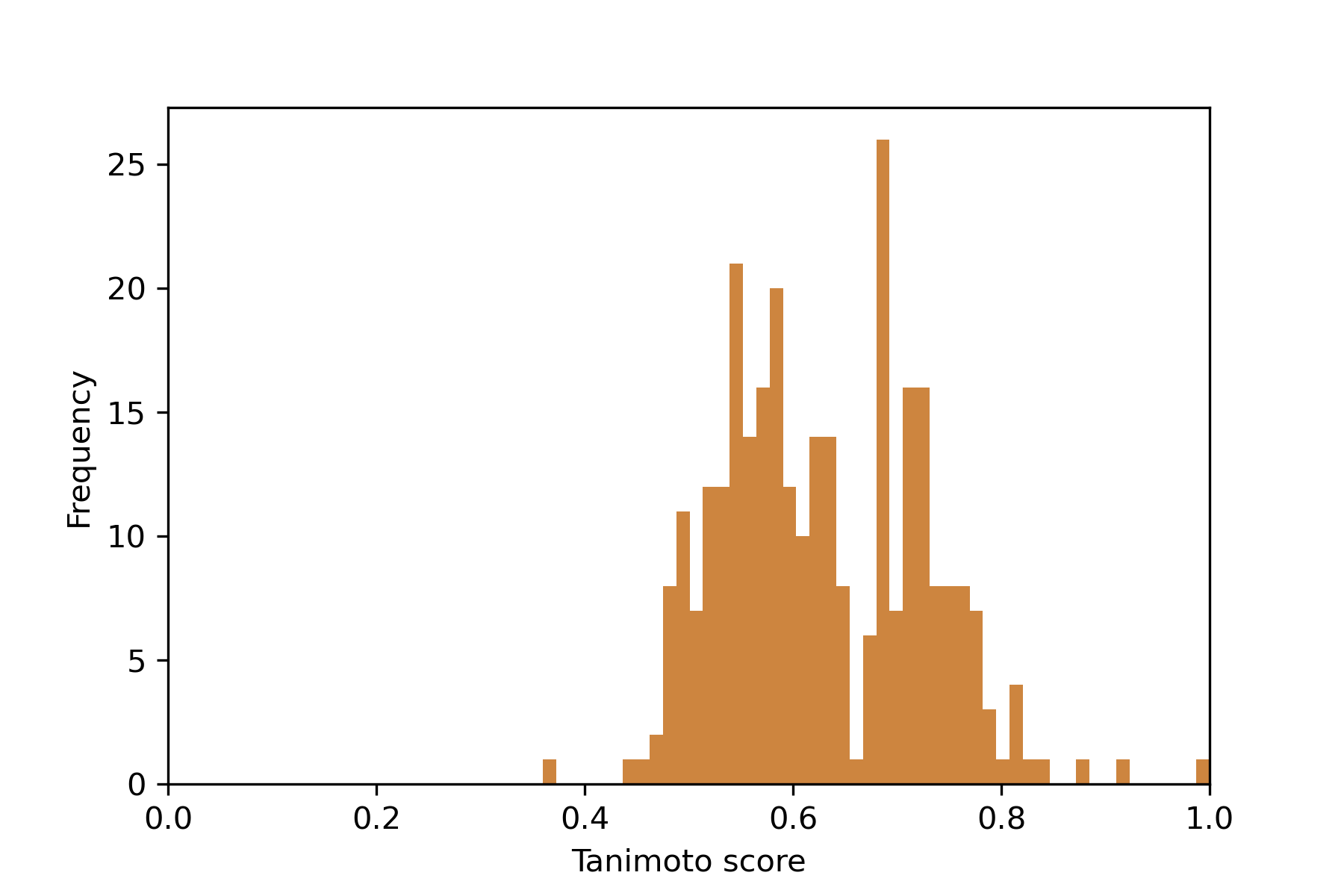

Supplement: Supplementary file 1 [file ijms-26-11481-s001.zip › FigureS1.png]
